# Roles of organokines in intervertebral disc homeostasis and degeneration

**DOI:** 10.3389/fendo.2024.1340625

**Published:** 2024-03-12

**Authors:** Yuxin He, Sheng Liu, Hui Lin, Fan Ding, Zengwu Shao, Liming Xiong

**Affiliations:** ^1^ Department of Orthopaedics, Union Hospital, Tongji Medical College, Huazhong University of Science and Technology, Wuhan, China; ^2^ Department of Orthopaedics, JingMen Central Hospital, Jingmen, China; ^3^ Hubei Minzu University, Enshi, China

**Keywords:** intervertebral disc homeostasis, intervertebral disc degeneration, organokines, organ crosstalk, signaling pathway

## Abstract

The intervertebral disc is not isolated from other tissues. Recently, abundant research has linked intervertebral disc homeostasis and degeneration to various systemic diseases, including obesity, metabolic syndrome, and diabetes. Organokines are a group of diverse factors named for the tissue of origin, including adipokines, osteokines, myokines, cardiokines, gastrointestinal hormones, and hepatokines. Through endocrine, paracrine, and autocrine mechanisms, organokines modulate energy homeostasis, oxidative stress, and metabolic balance in various tissues to mediate cross-organ communication. These molecules are involved in the regulation of cellular behavior, inflammation, and matrix metabolism under physiological and pathological conditions. In this review, we aimed to summarize the impact of organokines on disc homeostasis and degeneration and the underlying signaling mechanism. We focused on the regulatory mechanisms of organokines to provide a basis for the development of early diagnostic and therapeutic strategies for disc degeneration.

## 1 Introduction

Intervertebral disc degeneration (IVDD) is the main contributor to the development of low back pain, leading to a remarkable loss of disability-adjusted life years as well as a substantial economic burden on society (1, 2). Healthy discs are cartilaginous structures that contribute one-third of the spine height and act as “elastic cushions” providing essential support, absorbing mechanical stress through compression, and providing flexibility. At the core of discs lies the gel-like nucleus pulposus (NP), which is surrounded by a concentric layer-arranged annulus fibrosus (AF) and two semi-rigid thin cartilage endplates (CEPs) that lie beneath the adjacent vertebrae. Various biological processes, including inflammation modulation, prevention of neovascularization, cell homeostasis, and matrix metabolism balance, are essential for preserving the disc homeostasis (3). IVDD, which is characterized mainly by persistent inflammation and matrix metabolism imbalance, refers to the progressive deterioration of the disc structure, leading to disc herniation, disc height loss, and nerve compression (3). The therapeutic options for IVDD are limited due to a poor understanding of the underlying mechanisms.

The healthy disc is not isolated from other tissues, despite having long been known as a unique organ without blood vessels, nerves, or immune cell infiltration (4). An increasing number of studies have shown that organokines, the bioactive factors secreted by diverse tissues, may have a vital impact on disc homeostasis. The expression of organokines can be induced by several factors, including physical activity, diet, aging, and metabolic alterations like obesity and diabetes (5, 6). Through autocrine, paracrine, or endocrine mechanisms, organokines have been linked to several inflammatory diseases, such as rheumatoid arthritis (7, 8). However, the role of organokines in IVDD is not completely understood.

**Figure 1:**
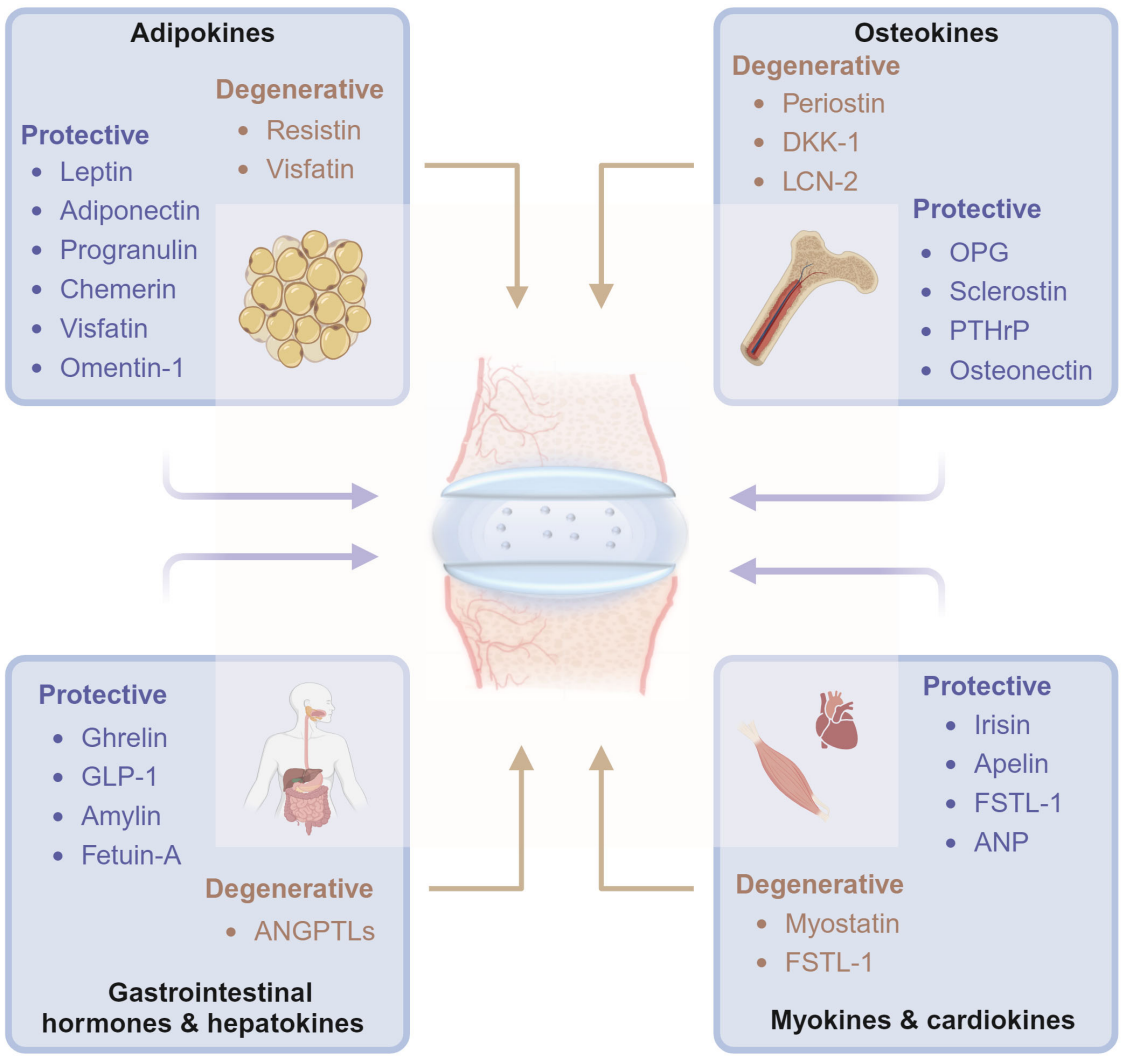
Diagrams illustrate that organokines, including adipokines, osteokines, myokines, cardiokines, gastrointestinal hormones and hepatokines, mediates the cross-organ regulation of disc homeostasis under physiological and pathological conditions from the major tissues of endocrinory ability. OPG, osteoprotegerin; DKK-1, dickkopf-1; PTHrP, Parathyroid Hormone-Related Protein; SPARC, Secreted protein acidic and rich in cysteine; BMPs, Bone morphogenetic proteins; ANP, atrial natriuretic peptide; GLP-1, Glucagon-like peptide-1; ANGPTLs, angiopoietin-like proteins; FSTL-1, Follistatin-like-1; LCN-2, lipocalin-2. Graphic elements were created using biorender.com.

In this review, we aimed to summarize the molecular and biochemical characteristics of organokines from specific tissues and their association with disc homeostasis and degeneration. The organokines treated in this review include adipokines, osteokines, myokines, cardiokines, gastrointestinal hormones, and hepatokines. Common hormones, growth factors, cytokines, and chemokines are excluded (**Figure 1**). Organokines play regulatory roles in cellular behavior, inflammation, and matrix metabolism in intervertebral disc homeostasis by binding to their receptors and activating downstream signaling pathways. We hope that this review will deepen the understanding of IVDD in the view of organ crosstalk and pave the way for the development of novel therapeutic interventions.

## 2 Adipokines

**Figure 2:**
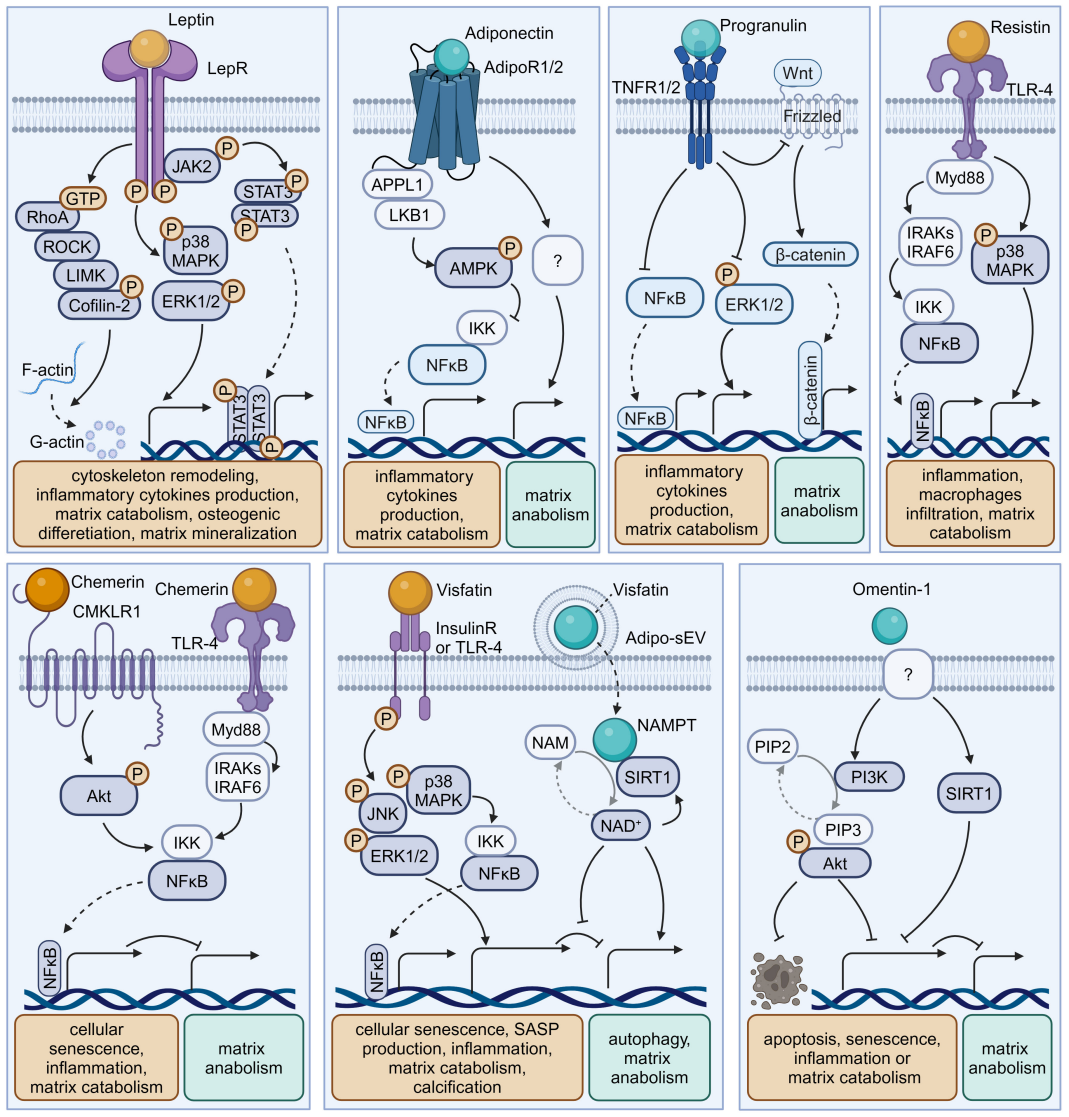
Schematic plots illustrate the signaling mechanism of various adipokines in intervertebral disc cells. Leptin, binding to LepR, activates JAK-2/STAT-3, MAPK, ERK1/2, RhoA/ROCK/LIMK/Cofilin-2 pathways, promoting disc degeneration. Adiponectin, binding to AdipoR1/2, activates AMPK, inhibiting the NF-κB pathway to exert a protective effect. Progranulin activates TNFR1/2, inhibiting NF-κB, ERK1/2, and Wnt/β-catenin pathways, providing a protective effect. Resistin, binding to TLR-4, inhibits NF-κB or MAPK pathways, exhibiting a protective effect. Chemerin, binding to CMKLR1 or TLR-4, activates the NF-κB pathway, promoting disc degeneration. Visfatin, binding to Insulin receptors (InsulinR) or TLR-4, activates JNK, MAPK, ERK1/2, and NF-κB pathways, promoting disc degeneration. However, the intracellular NAMPT activity of Visfatin delivered by Adipo-sEV could promote NAD^+^ biogenesis and SIRT activity, exerting a protective effect. Omentin-1 activates PI3K/Akt and SIRT1 pathways, providing a protective effect, though its receptor remains incompletely understood. Graphic elements were created using biorender.com.

Obesity, characterized by excessive adipose tissue, has been recognized as a significant risk factor for disc degeneration (9). In recent decades, adipose tissue has been considered as an endocrine organ that secretes various bioactive factors named adipokines (10) (**Figure 2**, **Table 1**). The cell-signaling proteins, such as leptin, adiponectin, and progranulin (PGRN) are secreted from adipose tissues and act like cytokines in the obesity-related impact on non-adipose tissues (33, 34). Research suggests that adipokine signaling is involved in the regulation of intervertebral disc homeostasis by several conditions, including disc tissue disruption by vertebral osteomyelitis, disc inflammation by ectopic adipose tissue infiltration, and osteonectin deletion-induced disc degeneration in mice (35–38).

**Table 1:** Main characteristics of adipokines modulating IVD homeostasis and degeneration.

Organokines (Receptors)	Target	Model	Signaling pathway	Cellular behavior or phenotype induced by organokines	Citation
Leptin (LepR)	NPC AFC	Exposure^a^	MAPK↑; PI3K/Akt↑; JAK-2/STAT-3↑	Inflammation (IL-6, TNF-α)↑; NO↑; Lactate↑; Catabolism (MMP-1, 9, 13; ADAMTS-4, 5)↑	(11–14)
	NPC	Exposure^a^	RhoA/ROCK/LIMK/Confilin-2↑	Cytoskeleton remodeling↑	(15)
	AFC	Exposure^a^	MAPK/ERK1/2↑	Differentiation (Col IX, MMP-13)↑	(16)
	CEP	Exposure^b^	ERK1/2↑; STAT-3↑	Matrix mineralization (RUNX-2)↑; Cartilage and chondrocyte↓; Disc height↓	(17)
	IVD	LepR KO^b^	NA	Proliferation↓; Differentiation↓; Disc height↓; Torsional strength↓	(18)
Resistin (TLR-4)	NPC AFC	Exposure^a^	MAPK↑; NF-κB↑	Infiltration of macrophages (CCL4)↑; Inflammation (NLRP3, caspase-1, IL-1β, IL-6, IL-8)↑; Catabolism (MMP-1, 3, 13; ADAMTS-5)↑	(19, 20)
Adiponectin (AdipoRs)	NPC	Exposure^a^	AMPK/NF-κB↑; TNF-α↓	Inflammation (TNF-α, IL-6)↓; Anabolism (Acan, Col II)↑; Catabolism (MMP-13, ADAMTS4)↓	(21)
	NP, AF	Exposure^b^	AMPK/NF-κB↑	AdipoR1↓, AdipoR2↓; Histological scores↓; DHI↑;	(21, 22)
Visfatin/NAMPT (Insulin receptor, TLR-4)	NPC CEPC	Adipo-sEV delivery^a,b^	SIRT1/NAD^+^↑	Senescence (p16)↓;,SASPs:(TNF-α, IL-6, IL-8)↓; Matrix mineralization (OCN, RUNX2)↓; Anabolism (Acan, Col II)↑; Catabolism (MMP-3, ADAMTS4)↓; Pfirrmann grade↓	(23)
	NPC	KD^a^;OE^a^	MAPK/NF-κB↑	Autophagy (Beclin-1, LC3B)↓; Inflammation (TNF-α, NLRP3)↑; Anabolism (Acan, Col II)↓; Catabolism (MMP-3,13; ADAMTS-4, 5)↑;	(24, 25)
	NP	Exposure^b^	MAPK↑; JNK/ERK1/2↑	Inflammation (IL-6)↑; Anabolism (Acan, Col II)↓; Catabolism (MMP-3)↑; Pfirrmann grade↑	(26)
Progranulin (TNFR1/2)	NPC	Exposure^a,b^	NA	Inflammation (MMP-13, COX-2, iNOS, IL-17) ↓; Chondro-staining density↑; Histological scores↓	(27)
	NPC NP	Analogue (Atsttrin)^a^; TNFR1/2 KO^b^	NA	Apoptosis↓; Catabolism (MMP-13)↓; Anabolism (Acan)↑; Histological scores↓; Pfirrmann grade↓	(28)
	NP, CEP, AF	PGRN KO^b^	NF-κB↓ Wnt/β-catenin↓	Inflammation (IL-17↓, IL-10↑)↓; Matrix mineralization (ALP, OCN, Osterix, BSP, Col I, AXIN2, RUNX2)↓; Anabolism (proteoglycan)↑; Catabolism (MMP-13, ADAMTS-5, 7, 12)↓; Pfirrmann grade↓	(29, 30)
Chemerin (CMKLR1, TLR-4)	NPC AFC	KD^a,b^; Exposure^a^	Akt↑; NF-kB↑	Inflammation (COX-2, IL-1β, IL-6, TNF-α)↑; Senescence (SA-β-gal, p53, p16)↑; Anabolism (Acan, Col II, SOX-9)↓; Catabolism (MMP-3, 9; ADAMTS-5)↑; Histological scores↑; DHI↓;	(31)
Omentin-1 (NA)	NPC	Exposure^b^	SIRT1↑	Senescence (SA-β-Gal, p16, p53)↓ Anabolism(Acan, Col II)↑; Catabolism (MMP-13, ADAMT-5)↓;	(32)

↑, increase; ↓, decrease; NA, not available; Exposure^a^, exposure in vitro; Exposure^b^, exposure in vivo; KD^a^, knock down in vitro; KD^b^, knock down in vivo; KO^b^, knock out in vivo; OE^a^, overexpression in vitro; Receptor activation^a^, receptor activation in vitro; Receptor activation^b^, receptor activation in vivo; LepR, leptin receptor; LRPs, low density lipoprotein-related proteins; TLR-4, toll-like receptor-4; IL, interleukin; Acan, aggrecan; Col II, type II collagen; Col lX, type lX collagen; OCN, osteocalcin; RUNX2, RUNX family transcription factor 2; STAT-3, signal transducers and activators of transcription 3; CCL4, C-C motif chemokine ligand-4; AdipoR, adiponectin receptor; DHI, disc height index; NF-κB, transcription factor-Κb; NLRP3, NLR family pyrin domain containing3; iNOS, inducible nitric oxide synthase; ALP, alkaline phosphatase; BSP, bone sialoprotein; AXIN2, axis inhibition protein 2; LC3, microtubule-associated protein 1A/1B-light chain 3; NAMPT, nicotinamide phosphoribosyl transferase; CMKLR1, chemokine-like receptor 1; SOX-9, SRY-box transcription factor -9; SIRT1, NAD-dependent deacetylase sirtuin-1.

### 2.1 Leptin

Leptin is a peptide hormone that is mainly synthesized in white adipose tissue and plays a regulatory role in energy metabolism and body weight. Beyond enhancing energy consumption in target cells, leptin can promote the production of pro-inflammatory cytokines, underlying the inflammatory and painful impacts of obesity. Both the leptin protein and the leptin receptor (LepR) have been detected in discs and are positively correlated with age and degeneration severity (11). While leptin can induce osteogenic differentiation in CEPs (17), the levels of leptin and its receptors increase with matrix metalloproteinase (MMP) and cytokine levels in the AF and NP of degenerative discs (12, 16, 39). Mechanistically, leptin can drive matrix catabolism via the Janus kinase-2 (JAK-2)/signal transducer and activator of transcription-3 (STAT-3) and mitogen-activated protein kinase (MAPK) pathways (13, 40). Additionally, leptin activates the ras homolog gene family member A (RhoA)/rho-associated coiled-coil containing protein kinase (ROCK) pathway and cytoskeletal remodeling in response to mechanical signals (15, 41). Although these findings suggest leptin has detrimental effects, whole-body leptin receptor knockout mice display delayed cellular proliferation and differentiation, elevated MMP-3 levels, and higher apoptosis rates, leading to IVDD (14, 18). Moreover, LepR has been identified as a lineage marker and fate modulator of notochord-derived cells at perinatal stages (42). Therefore, the potential fundamental role of leptin–LepR interactions in IVDD requires further exploration.

### 2.2 Adiponectin

Adiponectin, a glycoprotein that is uniquely expressed by adipocytes, could maintain energy balance and suppress inflammation or apoptosis in various tissues by binding to adiponectin receptors (AdipoR1/2). However, the role of adiponectin and AdipoRs in IVDD is unclear. Previous studies showed that adiponectin expression in degenerative discs was decreased or absent while AdipoR1 and AdipoR2 expression increased or decreased with the Pfirrmann grade of degenerative discs (22, 43). However, plasma adiponectin levels were found to be increased in IVDD patients (44). Recently, administration of the AdipoR agonist AdipoRon was found to effectively reduce the levels of the pro-inflammatory factor tumor necrosis factor α (TNF-α) and mitigate disc degeneration (21). In future research, the exact role of adiponectin–AdipoR interactions in IVDD needs to be clarified.

### 2.3 Progranulin

PGRN, a secreted glycoprotein that can be cleaved into granulins by enzymes like elastase, exerts anti-inflammatory effects and plays protective roles by enhancing cell proliferation and through interacting with TNF receptors (TNFRs) or other receptors. Although higher PGRN levels are associated with higher degeneration severity in IVDD patients, current evidence suggests a protective role for PGRN in disc degeneration and aging (3, 45). Knockdown of PGRN in aged mice accelerates disc degeneration by promoting matrix catabolism and cellular dysfunction in AF and CEPs (29). Mechanistically, PGRN competitively binds to TNFR-1, thereby inhibiting the expression of the pro-inflammatory factor interleukin-17 (IL-17) and inflammatory and catabolic pathways (30, 45). Moreover, PGRN promotes anabolism and the production of the anti-inflammatory factor IL-10 via binding to TNFR-2 (28, 30). Additionally, PGRN and its derivatives, like atsttrin, inhibit epoxide synthase-2, IL-6, IL-17, and MMP-13 production, thereby inhibiting IVDD progression (29, 30).

### 2.4 Resistin

Resistin is a cysteine-rich polypeptide that is secreted by white adipocytes and is involved in insulin resistance. In agreement with the devastating effect of diabetes on IVDs, recent research indicates resistin’s involvement in IVDD for its pro-inflammatory properties (46). By targeting Toll-like receptor-4 (TLR-4), resistin activates the nuclear factor kappa-light-chain-enhancer of activated B cells (NF-κB) signaling to increase the expression of the macrophage inflammatory protein chemokine C-C motif ligand 4 (CCL4), thereby fostering macrophage infiltration into discs (19). In addition, resistin triggers inflammatory cascades through the activation of the MAPK and NF-κB pathways, which increases the NLR family pyrin domain containing 3 protein (NLRP3) inflammasomes and the expression levels of IL-1β, IL-6, IL-8, and MMPs in discs (20, 47).

### 2.5 Chemerin

Chemerin is an obesity-associated adipokine and is involved in various processes including inflammation by interacting with chemokine-like receptor 1 (CMKLR1). The expression levels of chemerin and CMKLR1 are increased in degenerative NP tissues, especially those of obese individuals (31). Furthermore, the administration of chemerin results in inflammation and tissue degeneration, while CMKLR1 knockdown could slow the progression of needle-induced disc degeneration in rats (31). Furthermore, chemerin exerts pro-senescent and pro-inflammatory effects on NP cells through binding to TLR-4, a well-known receptor activating the NF-κB signaling cascade (31, 48).

### 2.6 Visfatin

Visfatin, identified as the extracellular form of nicotinamide-phosphate ribosyl transferase (NAMPT), has been known to mediate insulin resistance and inflammation via binding to the insulin receptor or the innate immune receptor TLR-4. Visfatin could induce IL-6 expression and disc degeneration by activating the MAPK pathway, which participates in the inflammatory response (26). In addition, pharmacological inhibition or knockdown of visfatin resulted in the maintenance of metabolism balance by enhancing autophagy in the presence of IL-1β (24). Interestingly, a recent study showed that NAMPT was delivered in small extracellular vesicles derived from adipocytes (Adipo-sMV) and mediated the protective impact of Adipo-sMV through increased nicotinamide adenine dinucleotide (NAD) and NAD-dependent deacetylase sirtuin-1 (SIRT1) activity in senescent NP and CEP cells (23). Considering the lack of a secretion signal sequence, visfatin/NAMPT may play a multifaceted role dependent on its location: serving as the rate‐limiting enzyme for NAD^+^ biosynthesis in the cytosol or binding receptors on the cellular surface after leakage into the extracellular space.

### 2.7 Omentin-1

Omentin-1, an anti-inflammatory adipokine, exhibits anti-inflammatory and antioxidant properties. Its expression level is inversely correlated with the progression of various diseases, including diabetes, obesity, and osteoarthritis (49, 50). Recent studies showed that omentin-1 could protect NP cells from ongoing senescence, inflammation, apoptosis, or matrix metabolism imbalance in the presence of IL-1β through activating SIRT1 or the phosphoinositide 3-kinase (PI3K)/protein kinase B (PKB, also known as Akt) signaling pathway (32, 49). Therefore, it is valuable to further investigate its *in vivo* therapeutic potential in IVDD treatment.

## 3 Osteokines

**Figure 3:**
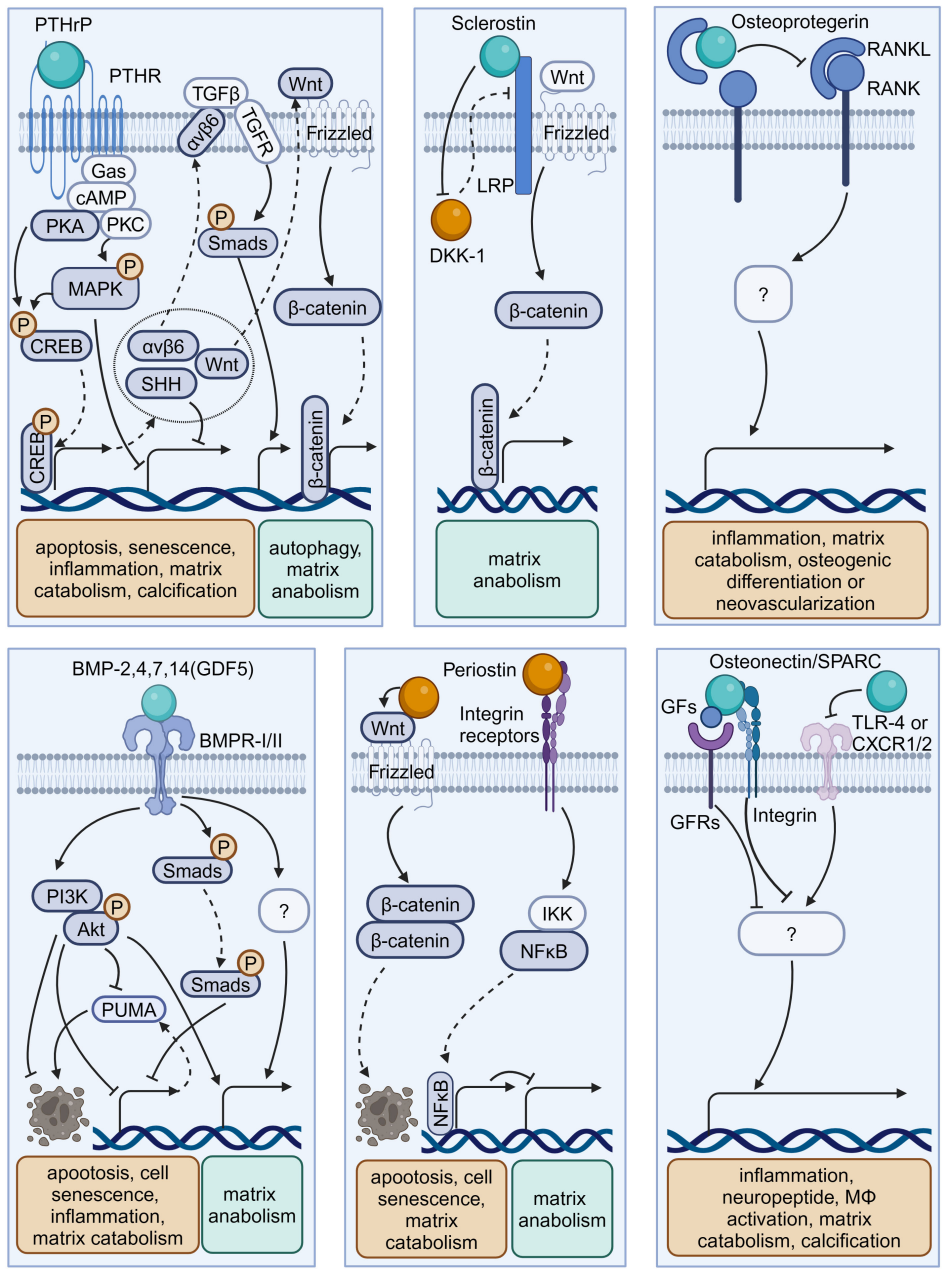
Schematic plots illustrate the signaling mechanism of various osteokines in intervertebral disc cells. PTHrP, binding to PTHR, could activate MAPK or PKA/CREB/Hedgehog pathways to protect disc from degeneration. Sclerostin binds to LRPs to activate Wnt/β-catenin pathway and matrix anabolism, while inhibits the expression of DKK-1 that inhibits Wnt/β-catenin pathway. Osteoprotegerin inhibits the RANK-RANKL interactions to protect disc from degeneration, while the intracellular signaling pathway is unknown. BMP-2,4,7,14(GDF5) binds BMPR-I/II to activate PI3K/Akt, Smads pathway and inhibit PUMA expression to protect disc from degeneration. Periostin could interact with Integrin receptors, NF-κB pathway and Wnt/β-catenin pathway to promote disc degeneration. Osteonectin/SPARC could modulate TLR-4, CXCR1/2, or GFs-GFRs interactions to protect disc from degeneration. Graphic elements were created using biorender.com.

Osteokines are a category of proteins predominantly secreted in bone and can have a significant influence on the homeostasis of bone and extraosseous organs (51–53). The interplay between bone homeostasis regulation and disc degeneration is becoming increasingly recognized. Indeed, the osteogenic potential of discs increases with the progression of degeneration, evidenced by elevated osteogenic differentiation of AF and CEP cells (54, 55). Then, intradiscal ectopic ossifications or calcifications can result in increased tissue stiffness, thereby provoking inflammation, disc degeneration, and low back pain (56–59). Additionally, structural alterations of vertebral bone, such as Modic changes (also known as magnetic resonance imaging [MRI] signal intensity changes in vertebral bone marrow) and vertebral osteoporosis, have been identified as associated with the development of IVDD (60, 61). Therefore, it is imperative to investigate the precise role of these osteokines (**Figure 3**, **Table 2**) in the pathophysiology of disc degeneration.

**Table 2:** Main characteristics of osteokines modulating IVD homeostasis and degeneration.

Organokines (Receptors)	Target	Model	Signaling pathway	Cellular behavior or phenotype induced by organokines	Citation
OPG (RANK/RANKL)	Disc cell	Exposure^a^	RANKL↓	Inflammation (IL-1β)↑; Catabolism (MMP-3, 13)↑	(62–64)
	CEP	OPG KO^b^	NA	Inflammation (IL-1β, IL-6, TNF-α)↑; Tissue remodeling (TRAP, Rank, MMP-9, Cathepsin K)↑; Chondrogenesis (cartilage area, growth plate thickness, aggrecan)↑; Neovascularization (VEGF-A, CD31, VE-cadherin, CD34)↑;	(65–67)
Sclerostin (LRPs)	NP	Sost KO^b^; Exposure^b^	Wnt/β-catenin↑	Matrix maturation (Col II, FOXA2, Osterix)↑; DDK-1↓; Matrix stiffness (proteoglycan↓; hydration↓)↑; DHI↓	(68)
PTHrP (PTH-1R)	NPC AFC	Analogue (PTH)^a^	mTOR↑; MAPK↑; PKA↑	Autophagy (Beclin-1, p62, LC3B)↑; Senescence (SA-β-gal)↓; Matrix mineralization (Acan↑, Col I↑, COLX↓, calcium release↓)↓	(69, 70)
	NPC CEP	Analogue (PTH)^a,b^	Wnt/β-catenin↑	Anabolism (Acan, Col II)↑; Catabolism (MMP3, 9)↓; Tissue remodeling (endplates calcification↓; micro-vessel density↓, porosity↑, thickness↑)↑; Histological score↓; DHI↑	(71, 72)
	NPC NP	Analogue (PTH)^a,b^	CREB/Sonic Hedgehog↑	Oxidative stress (SOD-1, 2)↓; Apoptosis (Caspase-3, 8, 9)↓; Inflammation (IL-1β, IL-6, TNF-α)↓	(73)
	IVD	PTH1R KO^b^; Analogue (PTH)^b^	Integrin αvβ6/TGF-β/CCN2↑	IVD volume↑; IVD height↑; MRI signal intensity↑	(74)
BMP-2,7 (BMPR-I/II)	NPC AFC NP	Exposure^ab^; KD^a^	PI3K/Akt↑; Puma↓	Apoptosis (Apaf-1, cleaved-caspase-3,9)↓; Senescence (SA-β-Gal, G0/G1 arrest, p16, p53)↓; Inflammation (IL-6 and TNF-α)↓; Anabolism (Acan, Col II, SOX-9)↑; Catabolism(MMP-13)↓; DHI↑;	(75–77)
Osteonectin/SPARC (CXCR1/2, TLR-4)	NP	SPARC KO^b^	NA	Inflammation (CXCL-1, 5)↓; Macrophage activation (ITGAM↓)↑; Endplate calcification↑; DHI↓	(78, 79)
	NP	SPARC KO^b^; Receptor inhibition^a^	NA	Inflammation (C3aR1, COX-2, CCL-7,19)↓; Catabolism (MMP-3, 13↓, TIMP1, 2↑)↓; Neutral zone stiffness↓;	(80)

↑, increase; ↓, decrease; NA, not available; Exposure^a^, exposure in vitro; Exposure^b^, exposure in vivo; KD^a^, konock down in vitro; KD^b^, knock down in vivo; KO^b^, knock out in vivo; Receptor activation^a^, receptor activation in vitro; Receptor activation^b^, receptor activation in vivo; RANK, receptor activator of NF-κB; RANKL, receptor activator of NF-κB ligand; PTH, parathyroid hormone; PTH1R, parathyroid hormone type 1 receptor; BMP, bone morphogenetic protein; BMPR, bone morphogenetic protein receptor; SPARC, secreted protein acidic and rich in cysteine; TRAP, tartrate-resistant acid phosphatase type 5; VEGF-A, vascular endothelial growth factor-A; VE-cadherin, vascular endothelial-cadherin; FOXA2, forkhead box protein a2; SOD, superoxide dismutase; CCN2, communication network factor-2; Apaf-1, apoptotic protease activating factor-1; CXCL, C-X-C motif ligand; ITGAM, integrin subunit alpha M; C3aR1, complement 3a receptor 1; TIMP,tissue inhibitor of matrix metalloproteinase.

### 3.1 Osteoprotegerin

OPG is a typical osteokine recognized as the regulator of bone mass and the receptor activator of NF-κB (RANK)/RANK ligand (RANKL) pathway, while it exists in various extraosseous tissues including discs. Various studies have shown a significant correlation between the levels of OPG in serum or disc samples and degeneration severity (62, 63, 81, 82). Further, OPG gene polymorphisms and increased OPG expression levels may contribute to IVDD development (81). OPG and RANK/RANKL expression could be upregulated with increased catabolism in AF, NP, or CEP cells exposed to acidic microenvironments or the inflammatory factor IL-1β (63, 64). However, OPG knockout results in osteoclast-mediated cartilage erosion, leading to disorganized alignment of CEPs, enhanced bone formation or neovascularization, and elevated inflammatory factors in mice (65–67). Thus, the multifaceted role of OPG in disc homeostasis highlights that further research is needed to elucidate its mechanism in IVD biology.

### 3.2 Sclerostin and Dickkopf-1

Sclerostin and DKK-1 are a pair of physical activity-related osteokines that competitively bind to the Wnt coreceptors lipoprotein receptor-related proteins (LRPs) and mediate the crosstalk between bone and other organs. Recently, sclerostin and DKK-1 have been shown to be involved in spinal pathological conditions, including spinal ligament ossification, spondylarthritis, and disc calcification (66, 67, 83). A recent study illustrated the compensatory increase in DKK-1 levels and the suppression of the Wnt/β-catenin pathway in sclerostin-depleted murine discs, and the administration of antibodies against sclerostin or DKK-1 exhibited beneficial effects on proteoglycan content, disc hydration, and height (84). Considering the complex role of Wnt signaling in disc development and degeneration, it is needed to clarify the exact roles and determinants of these Wnt inhibitors in IVDD (85).

### 3.3 Parathyroid hormone-related protein

PTHrP, first discovered in malignancy-associated hypercalcemia, has been recognized as an osteokine acting in a paracrine manner on bone and other tissues through binding to the PTH-1 receptor (PTH-1R). PTHrP is involved in intervertebral disc maturation and calcification, delays cellular mineralization and hypertrophy in Col IX knockout mice, and inhibits progressive kyphoscoliosis in fibroblast growth factor receptor-3 (FGFR-3) knockout mice (83–85). By enhancing Hedgehog, transforming growth factor beta (TGF-β), Wnt/β-catenin, mammalian target of rapamycin (mTOR), and MAPK/protein kinase A (PKA) signaling, PTH-1R activation by PTH administration plays a protective role in NP cell activity and disc homeostasis (69–72, 74). Considering the elevated PTH-1R expression in NP cells, the role of PTHrP–PTH-1R interactions in IVDD ought to be elucidated in future research (73, 86).

### 3.4 Bone morphogenetic proteins

BMPs are osteokines participating in the formation and maintenance of bone and various non-bone tissues, including cartilage (51, 87). Various studies confirmed the presence of BMPs, including BMP-2, 4, 7, and 14 (also known as growth differentiation factor -5 [GDF-5]), with their receptors (BMPR-I/II) in the IVD (88–92). Mechanistically, BMP-2 and BMP-7 activate various signaling pathways, including the Smad/Puma and PI3K/Akt signaling pathways, to inhibit NP cell apoptosis or senescence (75, 76). Additionally, GDF-5 deficiency in mice results in notable matrix abnormalities and disc degeneration, which could be substantially restored by treatment with recombinant human GDF5 (93, 94). Due to their anti-inflammatory and pro-regenerative effects, recombinant human BMPs are used for bone grafting in vertebral fusion surgery as well as disc tissue engineering (95, 96).

### 3.5 Osteonectin

Osteonectin, also known as secreted protein acidic and rich in cysteine (SPARC), is one of the most abundantly expressed non-collagenous proteins in mineralized tissues as well as non-mineralized tissues and orchestrates inflammation and tissue remodeling through binding to TLR-4, BMPRs, integrin receptors, and various growth factors. SPARC expression in human disc cells decreases with age and disc degeneration (97). Moreover, SPARC-deficient mice exhibit spontaneous disc degeneration and lower back pain, evidenced by an age-dependent increase in neuron markers like calcitonin gene-related peptide and Neuropeptide-Y within the discs and peripheral nerves (78, 98, 99). Additionally, these mice demonstrate a diminished lumbar neutral zone, increased spinal stiffness, and reduced spinal mobility (100). SPARC knockout results in elevated levels of inflammatory mediators and vascular endothelial growth factor, which can be mitigated by interventions like exercise and treatment with TAK-242 (a TLR-4 antagonist) or reparixin (an inhibitor of CXC chemokine receptors [CXCR1/2]) (80, 101–103). Therefore, SPARC is a promising target for preventing IVDD in modulating cell–matrix interactions and governing neural, immune, and inflammatory pathways (79, 104, 105).

### 3.6 Periostin

Periostin is a bone turnover-related osteokine that is highly expressed in collagen-rich tissue—including periosteum—and mediates tissue remodeling through binding to integrin receptors and proteoglycans. In human and rat discs, periostin levels gradually decrease from the outer AF to the central NP and increase with degeneration development (106–108). Mechanistically, periostin promotes NP cell apoptosis via the Wnt/β-catenin signaling pathway and cellular senescence via the NF-κB pathway, contributing to the development of IVDD (109, 110). Considering its role as a matricellular protein, further investigation is needed to elucidate whether periostin participates in the regulation of disc cell–matrix interactions (111).

### 3.7 Other potential osteokines

Lipocalin-2 (LCN-2), a glycoprotein secreted by osteoblasts and adipocytes, functions as a pro-inflammatory factor in obesity-related metabolic disorders, despite our limited understanding of the potential LCN-2 receptors (112, 113). A recent study suggested a correlation between LCN-2 and the expression of inflammation-related genes in human discs (114). Moreover, upregulated expression of LCN-2 has been validated to increase MMP-9 activity in AF cells (115). Considering that LCN-2 could function as a biomechanical and inflammatory sensor in bone–cartilage crosstalk, its specific role in IVDD needs to be elucidated (9).

Fibroblast growth factor-23 (FGF-23) is the first identified osteokine that can bind to the tyrosine kinase FGF receptors (FGFRs) to regulate phosphate and vitamin D metabolism (116). However, direct evidence linking FGF-23 to IVDD is currently lacking. Klotho, a crucial cofactor for FGF-23 in the activation of FGFRs, mitigates inflammation in NP cells and counteracts extracellular matrix degradation in IVDD (117, 118). Accordingly, the role of FGF-23 in IVD homeostasis, potentially analogous to that of Klotho, presents an intriguing avenue for further investigation.

## 4 Myokines and cardiokines

**Figure 4:**
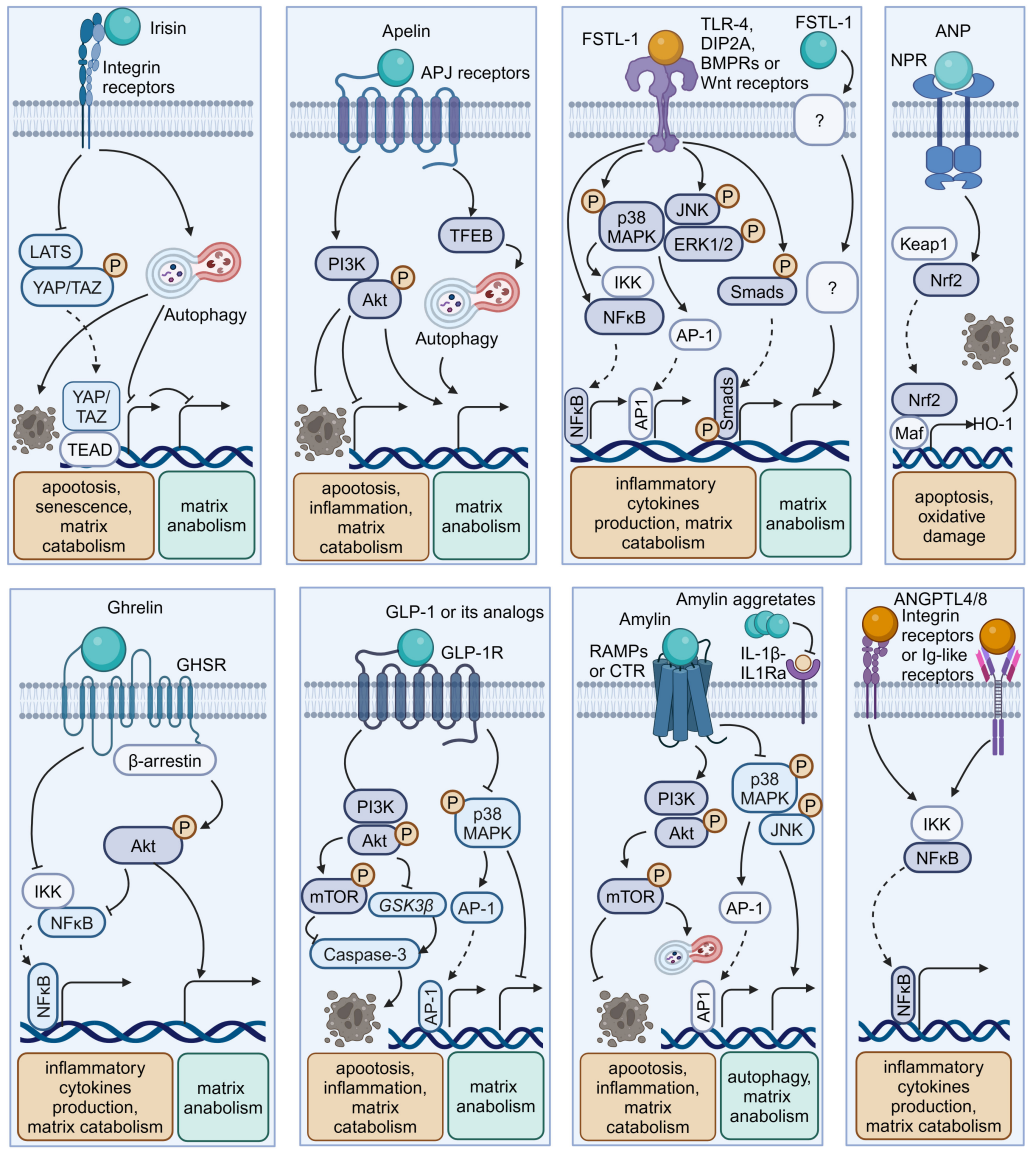
Schematic plots illustrate the signaling mechanism of other organokines in intervertebral disc cells. Irisin binds Integrin receptors to activate autophagy and inhibit LATS/YAP pathway to protect disc from degeneration. Apelin, binding APJ receptors to activate PI3K/Akt pathway and induce TFEB-mediated autophagy to protect disc from degeneration. FSTL-1, binding TLR-4, DIP2A, BMPRs or Wnt receptors, could activate MAPK, JNK, ERK1/2, NF-κB and Smad pathway to promote disc degeneration. Additionally, the FSTL-1 deficiency inhibit the maintenance of disc homeostasis. ANP binds NPR to protect cell from apoptosis and oxidative damage by activating NRF2/HO-1 pathway. Ghrelin binds GHSR to activate Akt and inhibit NF-κB pathways to protect disc from degeneration. GLP-1 or its analogs, binding to GLP-1R, activate PI3K/Akt/mTOR, PI3K/Akt/GSK3β, as well as inhibiting MAPK/AP-1 pathways to protect disc from degeneration. Amylin binds RAMPs or CTR to activate PI3K/Akt/mTOR pathway and inhibits MAPK/AP-1 pathways to protect disc from degeneration. Meanwhile, Amylin aggregates could inhibit IL-1β/IL1Ra interactions. ANGPTL4/8 could bind Integrin receptors or Ig-like receptors to activate NF-κB pathway to promote disc degeneration. Graphic elements were created using biorender.com.

Similar to adipose tissue and bone, skeletal muscle and cardiac muscle can function as endocrine organs and secrete tissue-specific hormones, termed myokines and cardiokines, respectively (119) (**Figure 4**, **Table 3**). It is well recognized that these molecules mediate cross-organ crosstalk beyond the muscle tissue itself and orchestrate the multi-tissue response to physical activity and other stress (112, 113, 119, 136). Given the emerging link between muscle activity and IVDD progression, the roles of myokines and cardiokines in IVDD deserve more attention and in-depth investigation.

**Table 3:** Main characteristics of other organokines modulating IVD homeostasis and degeneration.

Organokines (Receptors)	Target	Model	Signaling pathway	Cellular behavior or phenotype induced by organokines	Citation
Periostin (Wnt, Integrins)	NPC NP	Exposure^b^; KD^b^; Inhibitor^a^	Wnt/β-catenin↑; NF-κB↑	Apoptosis (Caspase-9, cleaved-caspase-3, Bcl-2, Bax)↑; Senescence (β-Gal, IL-1β, IL-6, IL-8)↑; Anabolism (Acan, Col II)↓; Catabolism (MMP-13)↑; Pfirrmann grade↑	(109, 110)
Irisin/FNDC5 (Integrin receptors)	NPC	FNDC5 KO^b^; OE^a,b^	AMPK/mTOR↑	Autophagy (p62, LC3B)↑; Senescence (SA-β-gal, p16)↓; Apoptosis (C-caspase-3)↓; Histological grades↓; DHI↑	(114)
	NPC	Exposure^a^	LATS/YAP/CTGF↑	Anabolism (Acan, Col II)↓; Catabolism (MMP-9, 13↑, ADAMTS-4, 5↑, TIMP-1, 3↓)↑	(115, 117)
Myostatin/GDF8 (ACVR1)	NPC	Receptor KD^a^	NA	Apoptosis↑; Inflammation (NF-α, IL-1β, IL-6)↑; Anabolism (Acan, Col II)↑	(120)
	IVD	Myostatin KO^b^	NA	Chondrogenesis (Col II, SOX-9, proteoglycan)↓; Endplate ossification↓	(121, 122)
Apelin (APJ)	NPC	Exposure^a^	TFEB↑	Autophagy (LC3B, p62)↑; Anabolism (Acan, Col II)↑	(123)
	NPC	Exposure^a^	PI3K/Akt↑	Apoptosis↓; Inflammation (IL-6, TNF-α)↓; Anabolism (Acan, Col II, SOX9)↑; Catabolism (MMP-3, 13)↓	(124)
FSTL-1 (TLR-4, etc.)	NPC	Exposure^a^	MAPK/ERK1/2↑; JNK↑; NF-κB↑	Inflammation (TNF-α, IL-1β, IL-6, COX-2, iNOS)↑; Catabolism (MMP-13)↑	(125, 126)
	NP	KD^b^	Smad1/5/8↑; ERK1/2↑; NF-κB↑	Inflammation (COX-2, iNOS, MMP-13, ADAMTS-5)↓; Cartilage area mean density↑	(127)
ANP (NPR)	CEPC	Exposure^a^	Nrf2/HO-1↑	Apoptosis (Bcl-2, Bax, C-caspase-3)↓; Oxidative Stress (MDA, SOD, NO)↓	(128)
Amylin/IAPP (RAMPs or CTR)	Disc cell IVD	Exposure^a^; KD^a^	IL-1β/IL-1Ra; PI3K/Akt/mTOR↑; MAPK/JNK↑	Apoptosis (Caspase-3↓, Fas/FasL↓, VDAC-1↓, cyto-C↓, Bax↓; Bcl-2↑)↓; Anabolism (Acan, Col II, SOX9)↑; Catabolism (MMP3, 9, 13; ADAMTS5)↓; Histological grades↓	(129–131)
Ghrelin (GHSR)	NP	Exposure^a,b^	NF-κB↓ Akt↑	Inflammations(MMP13, ADAMTS-5, TNF-α, iNOS)↓; Anabolism(Acan, Col II, SOX-9)↑; Pfirrmann grade↓	(132)
GLP-1 (GLP-1R)	NPC	Receptor activation^b^	MAPK/AP-1↓	Anabolism(Acan, Col II, SOX9)↑; Catabolism (ADAMTS5, MMP3, 13)↓; Histological scores↓; Pfirrmann grade↓	(133)
	NP	Receptor activation^a^	PI3K/Akt/mTOR& GSK3β↑	Apoptosis (Caspase-3)↓	(134, 135)

↑, increase; ↓, decrease; NA, not available; Exposure^a^, exposure in vitro; Exposure^b^, exposure in vivo; KD^a^, konock down in vitro; KD^b^, knock down in vivo; KO^b^, knock out in vivo; Receptor activation^a^, receptor activation in vitro; Receptor activation^b^, receptor activation in vivo; ACVR1, Activin receptors-1; NPR, Natriuretic peptide receptors; RAMPs, Receptor activity modifying proteins; CTR, C-terminal peptide; AP-1, activator protein 1; GSK3β, Glycogen synthase kinase-3 beta; VDAC-1, Voltage-dependent anion channel-1; Bcl-2, B cell CLL/lymphoma-2; Bax, Bcl-2-associated X protein.

### 4.1 Irisin

Irisin is a well-characterized myokine derived from fibronectin type III domain-containing protein 5 (FNDC5). It mediates the health benefits of exercise by binding with integrins. Exercise elevated irisin levels in plasma and NP tissue and FNDC5/irisin knockout abolished the protective effects of exercise against IVDD in a murine model (114). By activating autophagy or large tumor suppressor kinase (LATS)/yes-associated protein (YAP)/connective tissue growth factor (CTGF, also known as CCN2) signaling, irisin can help maintain cellular activity and matrix metabolism balance and inhibit inflammatory effects, thereby decelerating the progression of IVDD (115, 117).

### 4.2 Myostatin

Myostatin (also known as GDF8) functions as a negative regulator of skeletal muscle growth. It binds to activin receptors (ACVRs) and can be expressed in back muscles after IVD injury (118, 137). However, the role of myostatin in IVDD is incompletely understood. Myostatin plays an inhibitory role in cartilage formation and chondrocyte proliferation, and its serum levels exhibit a positive correlation with the severity of conditions such as osteoarthritis and rheumatoid arthritis (121, 138). Additionally, ACVR1 silencing reversed lipopolysaccharide-induced inflammation and matrix degradation in NP cells, implying the potential unfavorable impacts of ACVR1 activation by myostatin upon discs (120). However, earlier studies indicated the fundamental role of myostatin in disc homeostasis. Myostatin deficiency in mice resulted in increased muscle weight, accompanied by endplates ossification at the L4–L5 level and a notable reduction in proteoglycan content in the endplates and inner AF (122, 139). Therefore, more comprehensive research is needed to elucidate the potential mechanism underlying the multi-faceted role of myostatin–ACVRs interactions in IVDD.

### 4.3 Apelin

Apelin, identified as the endogenous ligand for the G-protein coupled receptor APJ, plays a regulatory role across diverse tissues including skeletal muscle and the cardiovascular system. Apelin and its receptor APJ are downregulated in degenerative NP tissue (123, 124). Moreover, administration of apelin results in suppressed matrix degradation, apoptosis, and inflammation in the presence of IL-1β and increased matrix anabolism in the presence of the oxidative stress inducer H_2_O_2_ (123, 124). Mechanistically, apelin enhances the PI3K/Akt pathway and transcription factor EB (TFEB)-mediated autophagy flux in NP cells (123, 124). Considering the significant role of apelin in exercise-induced benefits, exploring whether and how apelin participates in muscle–disc crosstalk is valuable (140).

### 4.4 Follistatin-like-1

Follistatin-like-1(FSTL-1) is a kind of myokine and cardiokine modulating immune responses, cell proliferation, and differentiation through binding to TLR-4, Wnt receptors, and various growth factors. FSTL-1 has an adverse effect on disc homeostasis, accompanied by increased concentrations in the serum of IVDD patients, discs of rats with IVDD, and the cerebrospinal fluid of dogs with IVDD (125, 141). Mechanistically, FSTL-1 promotes NP cell inflammation by activating the MAPK, Smads, or NF-κB signaling pathway (125, 141). Interestingly, the knockout of FSTL-1 during embryonic development leads to a decrease in vertebral cartilage and matrix anabolism, indicating its fundamental role in early IVD formation (142). Moreover, FSTL-1 may play diverse roles in disc development and maturation, given that it could mediate the differentiation of pre-cartilaginous stem cells into NP-like cells (143).

### 4.5 Atrial natriuretic peptide

As a typical cardiokine, ANP binds to natriuretic peptide receptors (NPRs) to induce diuretic, natriuretic, and vasodilating effects and regulate the renin–angiotensin–aldosterone system (144). NPR mutations can result in impaired cartilage development, potentially leading to secondary degenerative changes and suboptimal joint development (145, 146). Recent studies indicated that ANP inhibited oxidative damage and cell death in endplates by activating the nuclear factor erythroid 2-related factor 2 (Nrf2)/Heme oxygenase-1 (HO-1) signaling pathway (128). Additionally, given the presence and adverse effects of the local tissue renin–angiotensin system (tRAS) in discs, whether ANP has a protective impact via suppressing the tRAS in IVDD is an interesting avenue for future research (147–149).

## 5 Gastrointestinal hormones and hepatokines

In recent years, the interplay between the digestive system and disc homeostasis has received increasing attention, with a special focus on the role of the gut microbiota in the gastrointestinal endocrine system (150–153). It is worth noting that the endocrine functions of the digestive system facilitate complex inter-organ communication through various gastrointestinal hormones (such as Ghrelin and Amylin) and hepatokines (**Figure 4**, **Table 3**) (154, 155). Insight into how these endocrine factors influence disc physiology can expand our understanding of IVDD.

### 5.1 Ghrelin

Ghrelin is a circulating brain–gut peptide hormone that promotes growth hormone secretion via binding to the growth hormone secretagogue receptor (GHSR) and participates in the regulation of insulin resistance, obesity, and inflammation. Ghrelin was found present in the NP tissue, and ghrelin administration demonstrated a protective effect in a rabbit IVDD model (132). Mechanistically, ghrelin suppresses IL-1β-induced catabolism and inflammatory cytokine production by inhibiting the NF-κB pathway, while promoting anabolism via Akt signaling (132).

### 5.2 Glucagon-like peptide-1

Glucagon-like peptide-1(GLP-1), a peptide hormone secreted by intestinal L-cells, has broad pharmacological potential for managing type 2 diabetes mellitus and metabolic syndrome-related disorders by binding to its receptor -GLP-1R. GLP-1R activation leads to the inhibition of inflammation and apoptosis through downstream pathways including the PKA, PKC, and extracellular signal-regulated kinase 1/2 (ERK1/2) signaling pathways (156, 157). Notably, GLP-1R activation by liraglutide (a long-acting GLP-1 analog) has been shown to protect NP cells against hyperglycemia-induced apoptosis via the PI3K/Akt signaling pathway (134, 135). Administration of another GLP-1R agonist, exenatide, in discs promotes matrix synthesis and mitigates oxidative stress-induced matrix catabolism via inhibiting the activation of MAPK and activator protein-1 (AP-1) activity (133). Considering the therapeutic potential of GLP-1 activation, there is a need to further elucidate the role of endogenous GLP-1 in IVDD.

### 5.3 Amylin

Amylin, also known as islet amyloid polypeptide (IAPP), is a peptide that is predominantly secreted by pancreatic islet β-cells and participates in the development of diabetes through receptor activity-modifying proteins (RAMPs) or the calcitonin receptor (CTR) to inhibit insulin and glucagon secretion. During IVDD progression, amylin and its receptors are downregulated in the NP and AF cells, while amylin aggregates accumulate in NP tissues (129–131). Amylin overexpression in NP cells can maintain matrix metabolism balance and control the autophagy–apoptosis crosstalk by the PI3K/Akt/mTOR and MAPK signaling pathways (130). Meanwhile, these protective effects could be augmented by neutralizing IL-1β/IL-1 receptor antagonist (IL-1Ra) signaling induced by amylin aggregation (129). Furthermore, the amylin analog pramlintide showed the ability to relieve matrix metabolism impairment and enhance cell survival via a mitochondrial-dependent apoptotic pathway in NP cells (158). Additionally, amylin activates Akt/mTOR signaling to protect AF cells from death through the death receptor Fas/FasL and the mitochondrial-dependent apoptotic pathway (131).

### 5.4 Hepatokines

Hepatokines, such as angiopoietin-like proteins (ANGPTLs) and fetuin-A (also known as α2-HS-glycoprotein), are hormone-like proteins secreted by hepatocytes (159). ANGPTLs act as modulators of lipid metabolism, angiogenesis, and inflammation via binding to integrin receptors and immunoglobulin-like receptors. However, their roles in regulating disc homeostasis are poorly understood. Recent research illustrated the correlation between the upregulation of ANGPTL4/8 and the severity of disc degeneration (160, 161). Mechanistically, ANGPTL4/8 appears to promote matrix degradation and the production of inflammatory cytokines like TNF-α through the activation of the NF-κB signaling pathway (161, 162). Fetuin-A functions as an indirect inhibitor of ectopic mineralization and inflammation. Recent studies demonstrated that intra-articular injection of fetuin-A derivatives leads to improved osteoarthritis scores and mobility in a rat osteoarthritis model (163). Thus, it is worthwhile to explore the role of fetuin-A in IVDD.

## 6 Regulatory factors of organokines

Organokines, serving as the potential communicator between the extra discal tissues and the disc, participate in pathological processes such as cell death, inflammation, and matrix loss, thereby contributing to IVDD onset and progression. Current evidence suggests that the release and interactions of organokines could be regulated by multiple factors, making their impact challenging to quantify. Lifestyle factors such as exercise, diet, stress, sleep, and microbiome profoundly influence organokines production, affecting disc homeostasis and susceptibility to IVDD-related diseases (6, 164–166). Exercise, known as beneficial for IVD homeostasis, can modulate the release and activity of organ factors like irisin, ANGPTL4, osteocalcin, and adiponectin (113, 167). Notedly, acute exercise can fast change levels of myokines, hepatokines, osteokines, and immune cytokines, while long-term training alters baseline adipokines (113, 167). For instance, exercise normalizes leptin and lowers resistin, reducing inflammation and insulin resistance, which may help protect against IVDD (113). Considering the individual variability in response, further research is essential to explore pharmacological mimics of exercise on organokines modulation for IVDD treatments.

Diet type or pattern have potential protective effects on disc homeostasis and degeneration, taking Dietary supplements such as n-3 fatty acids (FAs) and bioactive dietary polyphenol preparations (BDPP) for example (168, 169). Interestingly, recent studies indicate that dietary patterns and types are closely related to adipokine secretion (170). Mediterranean, low-fat, and low-carbohydrate diets have been found associated with decreased levels of leptin and vaspin and increased adiponectin (170). Leptin and vaspin may adversely affect disc homeostasis maintenance, while the role of adiponectin remains controversial. Therefore, future research may focus on identifying whether the secretion type, quantity, and activity of organokines underly the links between diet types, patterns, or nutritional supplements and disc homeostasis.

## 7 Conclusion and future directions

A variety of organokines from adipose, bone, muscle, or digestive tissues play an adverse or protective role in intervertebral disc homeostasis. Most studies have focused on the impact on cells or tissue of single origin and have not considered overall disc or extra discal dynamics. Functional studies using cell cultures and animal models are encouraged to comprehensively evaluate the role of organokines in IVDD, especially cross-organ communication. The impact and detailed mechanisms of organokines-mediated interactions warrant further investigation under both physiological and pathological conditions.

Future research should prioritize developing pharmacological agents or biologics designed to modulate organokines activity, agonists or antagonists for receptors of organokines and inhibitors for organokines signaling pathways for potential clinical applications. Current investigations into the regulation of organokines by exercise, diet, and stress predominantly rely on *in vitro* or animal models. Moreover, it is essential to elucidate which organokines paly dominant roles on disc cell homeostasis and matrix metabolism balance. Consequently, future studies need to be more holistic, examining the impact of specific lifestyle choices on the entire spectrum of organokines, ideally assessing both local disc tissue and systemic levels. Given that many aspects of these molecules in humans remain under-explored or contentious—such as their *in vivo* half-life, protein binding in circulation, effective concentration in disc tissues, receptor interactions, and overall impact on disc health—clinical trials face a considerable journey ahead.

## Author contributions

YH: Data curation, Investigation, Visualization, Writing – original draft, Writing – review & editing. SL: Investigation, Visualization, Writing – original draft, Writing – review & editing. HL: Funding acquisition, Investigation, Resources, Writing – review & editing. FD: Conceptualization, Funding acquisition, Supervision, Writing – review & editing, Project administration. ZS: Conceptualization, Project administration, Resources, Supervision, Writing – review & editing. LX: Conceptualization, Project administration, Resources, Supervision, Writing – review & editing.

## Glossary

**Table:**

AdipoRs	Adiponectin receptors
ACVRs	Activin receptors
AdipoR-2	Adiponectin receptor-2
AdipoR-1	Adiponectin receptor-1
Adipo-sMV	Small extracellular vesicles derived from adipocytes
AF	Annulus fibrosus
AMPK	AMP-activated protein kinase
ANGPTLs	Angiopoietin-like proteins
ANP	Atrial natriuretic peptide
AP-1	Activator protein-1
BMPs	Bone morphogenetic proteins
CEP	Cartilage endplates
CMKLR1	Chemokine-like receptor 1
CTGF	Connective-tissue growth factor
CTR	Calcitonin receptor
DKK-1	Dickkopf-1
DM	Diabetes mellitus
ECM	Extracellular matrix
ERK	Extracellular signal-regulated kinase
FGF	Fibroblast growth factor
FGFRs	FGF receptors
FNDC5	Fibronectin type III domain-containing protein 5
GDF5	Growth differentiation Factor 5
GHSR	growth hormone secretagogue receptor
GLP-1	Glucagon-like peptide-1
GLP-1R	Glucagon-like peptide-1 receptor
HO-1	Heme oxygenase-1
IL-17	Interleukin-17
IL-6	Interleukin-6
IVD	Intervertebral disc
IVDD	Intervertebral disc degeneration
JNK	C-Jun N-terminal kinse
LATS	Latency-associated transcript
LCN-2	Lipocalin-2
lepR	Leptin receptor
LRPs	Lipoprotein receptor-related proteins
MAPK	Mitogen-activated protein kinase
MMP	Matrix metalloproteinase
MMP-13	Matrix metalloproteinase-13
MRI	Magnetic resonance imaging
mTOR	Mammalian TOR
NAD	Nicotinamide adenine dinucleotide
NAMPT	Nicotinamide-phosphate ribosyl transferase
NF-κB	Transcription factor-Kb
NLRP3	NLR family pyrin domain containing 3
NP	Nucleus pulposus
NPR	Natriuretic peptide receptors
Nrf2	Nuclear factor E2-related factor 2
OA	Osteoarthritis
OPG	Osteoprotegerin
PGRN	Progranulin
PI3K	Phosphoinositide 3-kinase
PKA	protein kinase A
PKC	protein kinase C
PTH	Parathyroid hormone
PTH1R	Parathyroid hormone 1 receptor
PTHrP	Parathyroid hormone Related Protein
RAMPs	Receptor activity modifying proteins
rhGDF-5	Recombinant human growth differentiation factor 5
SPARC	Secreted protein acidic and rich in cysteine
TGF-β	Transforming growth factor-β
TLR-4	Toll-like receptor 4
TNF	Tumor necrosis Factor
TNFR1	tumor necrosis factor receptors 1
TNFR2	tumor necrosis factor receptors 2
TNFRs	tumor necrosis factor receptors
TNF-α	Tumor necrosis Factor Alpha
tRAS	Tissue renin–angiotensin system
